# Projection of global burden and risk factors for aortic aneurysm – timely warning for greater emphasis on managing blood pressure

**DOI:** 10.1080/07853890.2022.2034932

**Published:** 2022-02-10

**Authors:** Xuewei Huang, Zhouxiang Wang, Zhengjun Shen, Fang Lei, Ye-Mao Liu, Ze Chen, Juan-Juan Qin, Hui Liu, Yan-Xiao Ji, Peng Zhang, Xiao-Jing Zhang, Juan Yang, Jingjing Cai, Zhi-Gang She, Hongliang Li

**Affiliations:** a Department of Cardiology, Renmin Hospital, School of Basic Medical Science, Wuhan University, Wuhan, China; b Institute of Model Animal, Wuhan University, Wuhan, China; c Medical Science Research Center, Zhongnan Hospital of Wuhan University, Wuhan, China; d Huanggang Institute of Translation Medicine, Huanggang, China; e Department of Cardiology, Center Hospital of Huanggang, Huanggang, China; f Department of Cardiology, Zhongnan Hospital of Wuhan University, Wuhan, China; g Department of Gastroenterology, Tongren Hospital of Wuhan University & Wuhan Third Hospital, Wuhan, China; h Department of Cardiology, The Third Xiangya Hospital, Central South University, Changsha, China

**Keywords:** Aortic aneurysm, global burden of disease, mortality rate, prediction, risk factor/global assessment

## Abstract

**Rationale:**

Aortic aneurysm (AA) is a serious condition that largely increases the risk of aortic dissection and sudden death. Exploring the global burden of disease and changes in risk factors for AA is essential for public health policy development.

**Objective:**

To project the death burden from AA and its attributable risk factors in the following decade based on the epidemiological data over the past 30 years.

**Methods and Results:**

We analysed the death burden of AA and trends of four risk factors from 1990–2019 using the updated 2019 Global Burden of Disease study database by Joinpoint regression analysis. Furthermore, we project the AA-related death burden for the next decade using the Bayesian age-period-cohort model. This study discovered that the global burden of death attributable to AA began to increase after decreasing for two decades. This upward trend will continue in the subsequent decade (average annual percent change: 0.318%, 95% CI: 0.288 to 0.348). Meanwhile, the disease burdens in all economic regions except high-middle socio-demographic index (SDI) regions will continuously increase in the next decade, with the fastest acceleration in the low-middle SDI region (average annual percent change: 1.183%, 95% CI: 1.166 to 1.200). Notably, high systolic blood pressure will surpass the contribution of smoking to become the most important risk factor for mortality due to AA.

**Conclusion:**

This study discovered a rebounding trend in the aortic aneurysm-related death burden globally. High systolic blood pressure will be the top risk factor attributed to death from AA. Therefore, it should be considered as the first-degree risk factor in the guidance of AA management and criteria for population-based screening programs.

**Key messages**
- The death burden of aortic aneurysms is beginning to rebound globally, and the trend will continue for the next decade.
- High systolic blood pressure will replace smoking as the most important risk factor associated with aortic aneurysm death.

## Introduction

Aortic aneurysm (AA) is a disease characterized by localized irreversible full-thickness dilation of the aorta, which is a severe condition that largely increases the risk of aortic dissection and sudden death [1]. With advances in treatments and declining smoking rates, the prognosis of AA has improved significantly in developed countries over the past decades [2–5]. However, as observed by the most recent analysis using 1990–2017 Global Burden of Disease Study (GBD) data, the decreasing mortality trend due to AA in the past few years has begun to plateau worldwide. AA-related mortality has increased in many regions, such as Central Asia, North Africa, and Central and Eastern Europe [6,7]. As an important cardiovascular disease, the global burden of AA may further increase due to population ageing and the proliferation of other important behavioural and metabolic risk factors, such as smoking, hypertension, non-alcoholic fatty liver disease, and hyperlipidaemia, especially in developing countries [8–10]. To address this serious disease, clarifying the current and future burdens of AA is imperative. Understanding the changes in and control of major risk factors for AA-related mortality is essential from disease prevention and control perspectives.

In this study, we constructed a Bayesian age-period-cohort model (BAPC) to project the death burden of AA from 2020–2030 based on updated 2019 GBD data. Moreover, we analysed changes in the contributions of major risk factors for AA mortality across sex and different regions. This knowledge is essential for developing of effective AA prevention and control strategies.

## Methods

### Data source

In this study, data on the death burden of AA from 1990–2019 were obtained from the updated 2019 GBD database (http://ghdx.healthdata.org/gbd-results-tool). As a worldwide well-known health database, GBD provides epidemiological data on dozens of diseases in 204 countries or regions. We acquired data on numbers of deaths, mortality rate, the age-standardized mortality rate (ASMR), and corresponding 95% uncertainty intervals (UI) associated with AA by region and sex for people aged 15 years and older. According to GBD 2019, the data of AA death burden comes from multiple relevant data sources, including civil registration and vital statistics, disease registries, health service use, and other sources. Everyone can access the data sources for each disease in each region by visiting the GBD website (http://ghdx.healthdata.org/gbd-2019/data-input-sources). The database also provides data on four risk factors associated with AA-related mortality: smoking, high systolic blood pressure (HSBP), diet high in sodium, and lead exposure. The data sources for risk factors in GBD 2019 were obtained from published literature, household survey reports (e.g. NHANES). The details of methodologies related to calculating indicators for the 2019 GBD have been described elsewhere [8,11]. Both GBD 2019 and this study are compliant with the Guidelines for Accurate and Transparent Health Estimates Reporting [12].

### Definitions

In the 2019 GBD database, AA includes thoracic (TAA) and abdominal aortic aneurysm (AAA); the two are not differentiated. Smoking was defined as the current daily or occasional use of any tobacco product. The theoretical minimum risk exposure level (TMREL) for HSBP was defined as 110 to 115 mmHg. Diet high in sodium was defined as a mean 24-hour urinary sodium excretion greater than 3 grams. The TMREL for lead exposure was two micrograms per decilitre of blood.

### Statistical analysis

To quantify the changes in the observed trends of AA, we used Joinpoint regression to analyse changes in trends across regions and by sex over the past 30 years. Joinpoint regression allows analysis of trends in disease over time and is more accurate than traditional linear fits, the significance test uses a Monte Carlo Permutation method, and *P*-values less than 0.05 will be considered significant [13]. The maximum number of Joinpoints was set to 5 in this study. Meanwhile, we calculated the average annual percent change (AAPC) to describe the magnitude of changes in the ASMR of AA. The calculated AAPC and the annual percent change (APC) within each trend segment, as compared to 0 to determine statistical significance.

Using the age-period-cohort model, the trends of AA-associated disease burden can be depicted and predicted, considering the impacts from age, periods, and cohorts. In detail, the age effect is the impact of age on disease occurrence. Differences in the risk of disease occurrence among subjects of the same age but at different periods can be considered the effect of period effects, such as advances in disease screening and treatment. The cohort effect is the effect of long-term exposure to risk factors or lifestyle habits on the risk of disease in subjects of the same birth cohort. Mortality due to AA is closely related to age. The increasing age of the population over the past 30 years has been accompanied by significant changes in AA-related risk factors and treatment. These changes may have an impact on the disease burden of AA. The age-period-cohort model allows for the analysis of changes in disease trends while controlling for age, period, and cohort effects. However, covariance among the three effects leads to the problem of unidentifiability in the classical age-period-cohort model. The Bayesian age-period-cohort model (BAPC) avoids this problem by including random effects, we completed the predictions using the BAPC package in R. The details have been explained elsewhere [14]. For prediction analysis at the national and regional levels, we used population data provided by the United Nations Economic and Social Council, which were available for a total of 187 countries and regions (https://population.un.org/wpp/Download/Standard/Population/).

To verify the prediction accuracy of the BAPC model, we selected the global and three regions (high SDI, Central Asia, and middle SDI) that showed different ASMR trends over the past 30 years, and the available data were divided into a training set (1990–2013) and a validation set (2014–2019), and the prediction results obtained using the training set were compared with those of the validation set and evaluated using the mean absolute percentage error

$$\text{mean absolute percentage error}=\frac{\sum \left((\left|\mathrm{Observed}-\mathrm{Projection}|∕|\mathrm{Observed}\right|)\times 100\%\right)}{\mathrm{Projection}\;\mathrm{years}}.$$

The results ranged from 2% to 6% (global: 3.70%, high SDI: 5.80%, Central Asia: 1.92%, middle SDI: 2.41%). To ensure the accuracy of the prediction, we excluded countries with abnormal prediction results due to a small number of deaths or low mortality rates when making predictions at the national and regional levels, and the burden of aortic aneurysm disease for 1990–2019 for these excluded countries or regions is displayed in Supplementary Table III. We finally included 150 countries or regions for prediction.

Joinpoint regression and AAPC calculations were performed using Joinpoint software (Version 4.9.0.0, Statistical Methodology and Applications Branch and Data Modelling Branch, National Cancer Institute, Bethesda, MD, USA), the other statistical analyses and data visualization were performed using R (Version 4.0.0, R Foundation for Statistical Computing, Vienna, Austria).

## Results

### The AA-related death burden has begun to rebound

**Figure 1.**
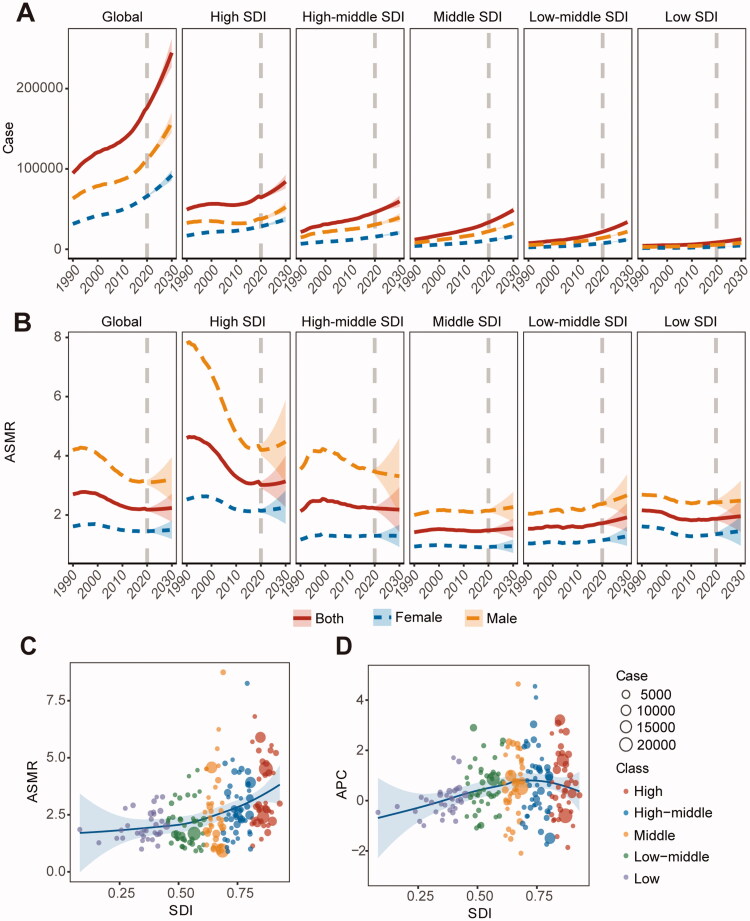
Aortic aneurysm (AA) death burden in different regions over the past 30 years and the next decade. (A) Trends in the number of death cases by gender in the different regions, 1990–2030. (B) Trends in age-standardized mortality rate (ASMR) by gender in the different areas from 1990–2030. (C) Relationship between ASMR in 2019 and socio-demographic index (SDI). (D) Relationship between annual percent change (APC) of ASMR for the most recent segment divided by Joinpoint regression analysis and SDI.

The changes in AA death burden over the past 30 years are shown in Figure 1(A,B). The number of deaths due to AA had increasing trends in all SDI regions over the past 30 years. Globally, the number of deaths increased by 82.1%, reaching 172,426, in 2019 compared with 1990. The global ASMR for AA began to increase in 2017–2019 (2017–2019, APC: 0.527%, 95% CI: −0.046% to 1.103%). Although this increasing trend was not statistically significant, it marked the end of a declining trend in AA mortality since 1994 (Supplementary Table I). Among the sociodemographic index (SDI) regions, all regions, except high-middle SDI regions, showed a significant increasing trend in the ASMR in the recent three years (Supplementary Table I). Notably, although the ASMR of AA increased with an increasing level of social development, low-middle to high-middle SDI areas have experienced the most rapid increases in ASMR recently (Figure 1(C,D)). The AA death burdens at the national and regional levels are shown in Supplementary Table III.

**Figure 2.**
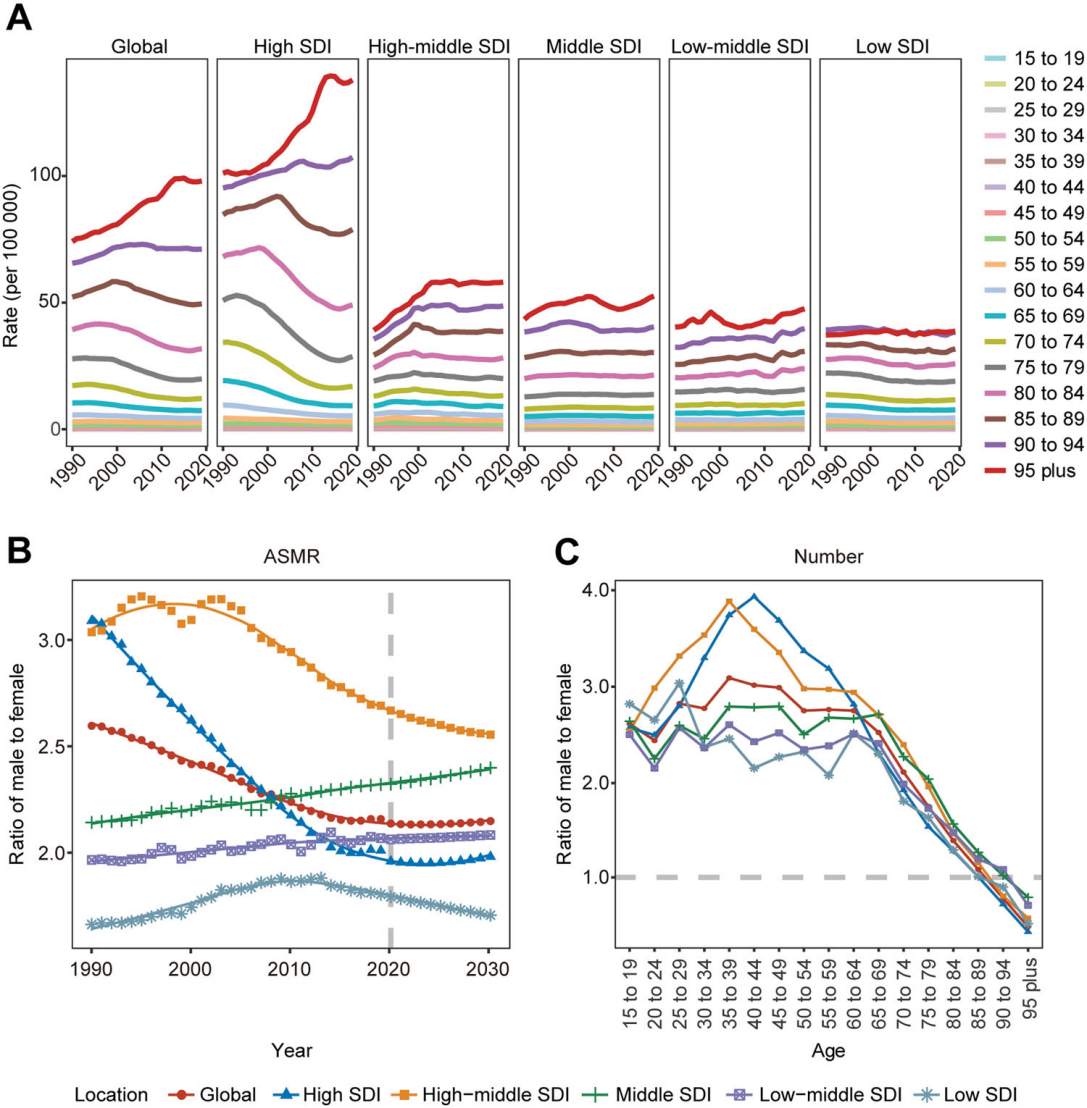
Age and sex differences in the burden of aortic aneurysm (AA) death. (A) Mortality in AA at different ages. (B) The ratio of AA age-standardized mortality rates (ASMR) in males to females. (C) The ratio of AA deaths by age in males to females. SDI: socio-demographic index.

In addition to the level of social development and geography, the death burden of AA differed significantly between age groups and sexes. According to the age group (Figure 2(A)), the death burden of AA among individuals aged over 70 years increased in recent years, particularly in high-SDI regions. For sex, the death burden was significantly higher in men than in women, but the sex difference in the global death burden of AA has narrowed over the past 30 years (Figure 2(B)). The burden of AA-related deaths between the sexes differed among age groups (Figure 2(C)). Notably, the ratio of men to women in deaths decreased rapidly after 65.

### The AA disease burden will continue increasing in the next decade

**Figure 3.**
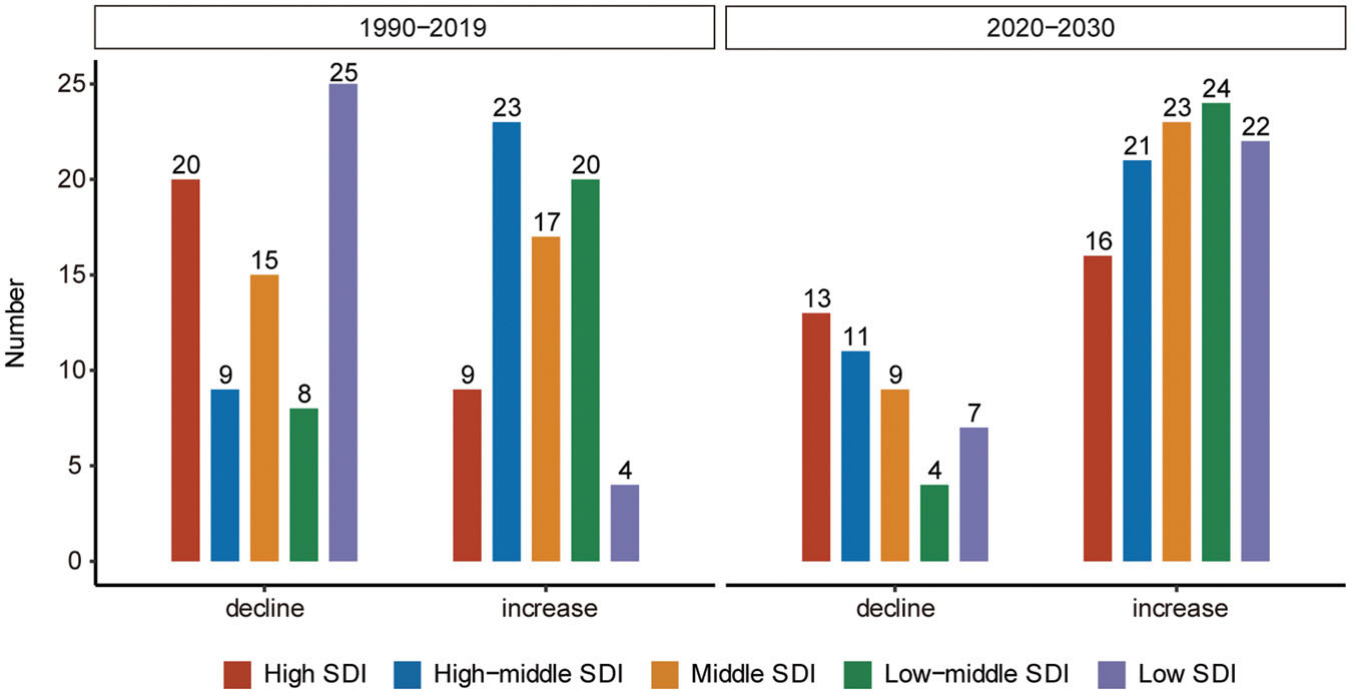
The number of countries or regions with increasing or decreasing age-standardized mortality rate of aortic aneurysms in the past 30 years compared with the next 10 years among the 150 countries or regions where the projections were conducted. SDI: socio-demographic index.

From 2020 to 2030, the number of AA-related deaths will continue increasing in all regions, with a 42% increase to 244,685 deaths in 2030 compared to that in 2019. Moreover, the global ASMR will also follow an increasing trend (AAPC: 0.318%, 95% CI: 0.288% to 0.348%), but it will vary by region (Supplementary Table II). In the next decade, low-middle SDI regions will experience the fastest increase (AAPC: 1.183%, 95% CI: 1.166% to 1.200%), while high-middle SDI regions will experience a decline in the ASMR (AAPC: −0.049%, 95% CI: −0.080% to −0.010%). In 2030, high SDI regions will still have the highest ASMR (3.134 per 100,000 population, 95% CI: 2.264 to 4.005) and middle SDI regions will have the lowest (1.550 per 100,000 population, 95% CI: 1.220 to 1.879). Of the 150 countries and regions included in the forecast, 106 and 44 countries will have increase and decrease trends, respectively, among which 51 will experience changes from decrease to increase trends (low SDI: 18, high SDI: 13, remaining: 20), while 55 continue to increase (low-middle SDI: 19, high-middle SDI: 15, remaining: 21) (Figure 3). The fastest increases will occur in Bangladesh (AAPC: 4.662%, 95% CI: 4.650% to 4.675%). Among the countries with more than 100 deaths, the fastest decline will occur in Ecuador. In 2030, Armenia will have the highest ASMR (10.020 per 100,000 population, 95% CI: 4.390 to 15.650), while India will have the highest number of deaths (28,942, 95% CI: 25,404 to 32,481) (Supplementary Table III).

In 2020–2030, the ASMR and numbers of deaths of AA will show increasing trends in both men and women globally. This death burden in men will remain higher than that in women (Figure 1(A,B)). The death number will continue rising in both sexes in each SDI region. For ASMR, only males in high-middle SDI regions show a decreasing trend (Supplementary Table II). The global differences between men and women will remain stable in the next decade, but regional differences will still be evident (Figure 2(B)).

### High systolic blood pressure will replace smoking as the most important risk factor for AA-related mortality

**Figure 4.**
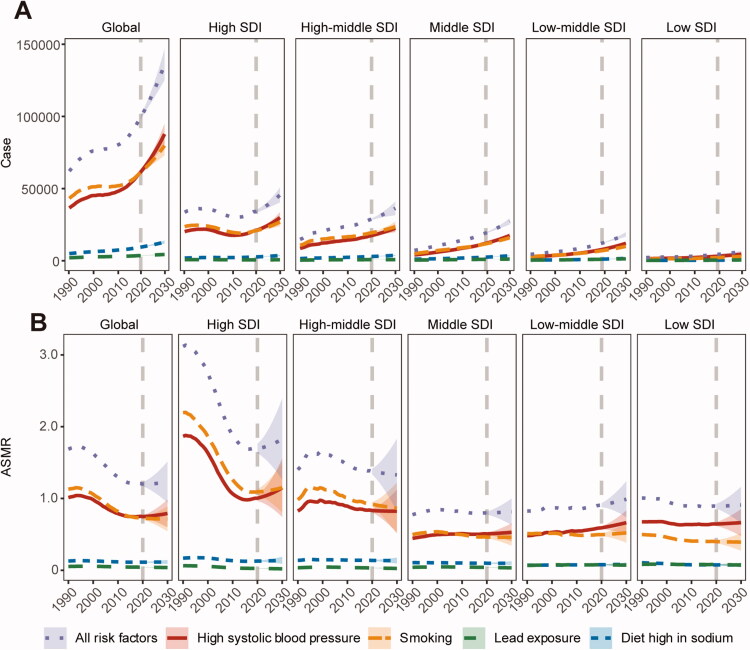
Changes in risk factors associated with aortic aneurysm (AA) deaths in different regions. (A) Trends in the number of deaths related to AA risk factors in the different regions from 1990–2030. (B) Trends in age-standardized mortality rate (ASMR) associated with AA risk factors in the different regions from 1990–2030. All, all risk factors; Sodium, a diet high in sodium; HSBP: high systolic blood pressure; Lead: lead exposure; SDI: socio-demographic index.

Smoking and HSBP are the two most important risk factors that increase AA mortality. Over the past three decades, the death burden associated with these two risk factors has decreased due to the control of smoking and HSBP in developed countries, which has also driven down the global ASMR of AA over the same period. However, in the last decade, the decline in smoking and HSBP associated ASMR tended to stop and even increased in some regions, while the number of deaths showed a rapid increase (Figure 4).

For this reason, we performed a predictive analysis of the changes in risk factors associated with AA deaths. We found that HSBP will overtake smoking as the most important risk factor for AA death. The number of AA deaths related to both smoking and high systolic blood pressure is increasing over the next decade. The increase in HSBP exceeds that of smoking, a trend similar across SDI regions (Figure 4(A)). In terms of ASMR, high systolic blood pressure has replaced smoking as the most critical risk factor associated with AA deaths by 2019 globally. The difference between the HSBP-attributable and smoking-attributable AA-related deaths will continue increasing in the next decade, which is largely contributed to the increased burden from HSBP (Figure 4(B)).

**Figure 5.**
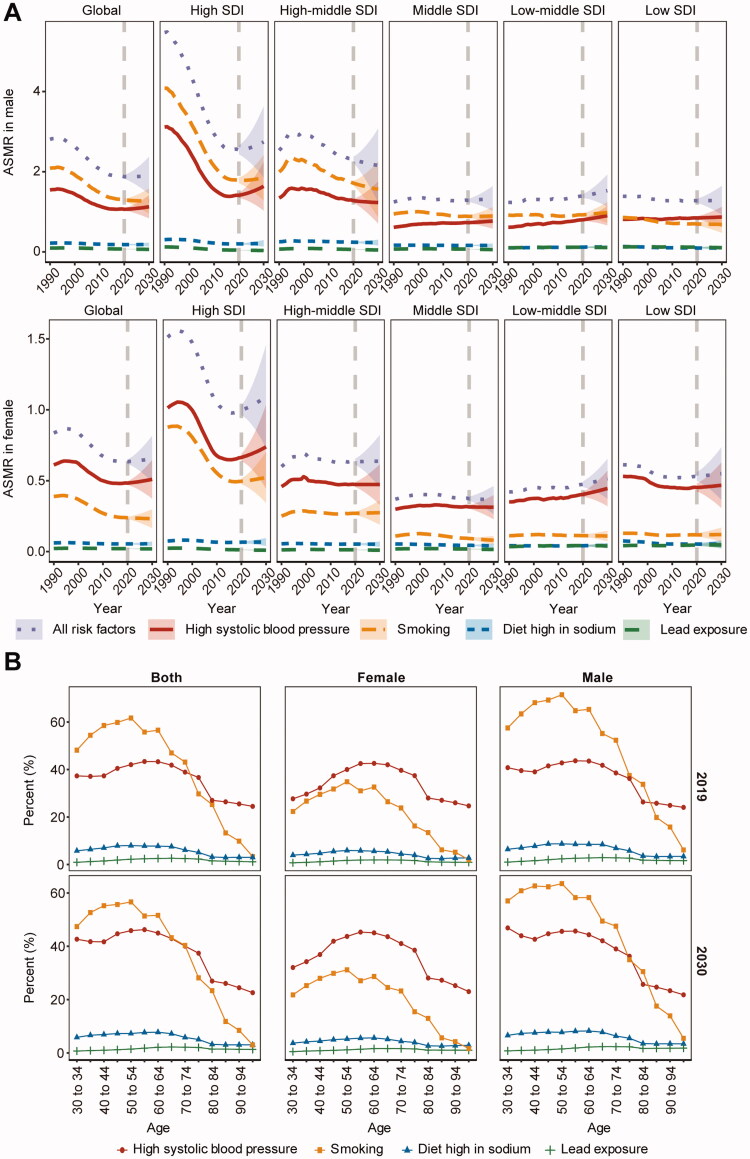
Differences in risk factors associated with aortic aneurysm (AA) death by gender and age. (A) Sex differences in different regions from 1990–2030. (B) Age differences in 2019 and 2030. ASMR, age-standardized mortality rate; Percent, changes in the proportion of AA deaths attributable to risk factors; SDI: socio-demographic index.

The burden of AA death associated with smoking and high systolic blood pressure differs significantly between men and women (Figure 5(A)). For men, smoking remains the predominant risk factor, but death burden from HSBP has surpassed smoking as the predominant risk factor in low SDI areas. By 2030, smoking will still be the predominant risk factor for men, but the gap between it and HSBP will narrow further. For women, HSBP has been the predominant risk factor in the past three decades, and the difference between HSBP and smoking has increased, a trend that will continue in the next decade.

The distribution of smoking and HSBP associated burden differed considerably by age (Figure 5(B)). Overall, smoking was the predominant risk factor for AA-related death in people aged less than 75 years, while in those aged more than 75 years, the risk associated with smoking was less than that associated with HSBP. The distribution in men was largely consistent with the overall trend. But HSBP was the predominant risk factor in all age groups of women. This difference between sex will remain in the next decade.

## Discussion

Using the most current 2019 GBD data, this study discovered that mortality due to AA began to increase in recent two years after decreasing for two decades globally. According to model projections, the increase in disease burden will continue in the following decade. Notably, HSBP, a dominant risk factor contributing to AA mortality, has not controlled well. Therefore, the contribution of HSBP will surpass the contribution of smoking and is predicted to be the most important risk factor contributing to mortality due to AA in the next 10 years. The findings of this study serve as a timely warning to enhance AA prevention and management efforts; they also provide a reference for the development of effective AA-prevention strategies.

The prevalence rates of abdominal aortic aneurysm (AAA) reported in most studies have shown decreasing trends in developed countries, and the prevalence of thoracic aortic aneurysm (TAA) may be increasing, but the prognosis has also improved [2,3,15–19]. Similarly, we found that high SDI regions have experienced a significant decrease in AA-related mortality over the past 20 years. However, our study suggests that AA-related mortality has increased in the recent three years and will continue increasing in the future. A study of trends in mortality from abdominal aortic aneurysms covering 17 countries showed an accelerating downward trend in mortality from abdominal aortic aneurysms as of 2015, but this study included fewer countries and mainly developed countries [20]. A Swedish population-based screening study observed a steady increase in the incidence of AAA among older men aged 65–99 years up to 2010, but they attributed the increase in the rate of diagnostic testing rather than a true increase in the incidence of AAA [4]. In contrast to their study, our study was based on AA-related mortality rather than AA incidence. We observed an increase in AA-related mortality in the context of declining autopsy rates in recent years; we believe this reflects the increasing burden of AA-related deaths.

We found that AA-related mortality tended to increase with the level of social development. Because the development of AA is age-related, this trend may be explained to some extent by the more severe ageing of populations in developed countries [21]. However, we also found that even though there were significant differences in demographic characteristics and common risk factors between high-middle and low SDI regions, the mortality rates were similar and significantly lower in all age groups in those regions than in high SDI regions. Considering that the majority of AA is asymptomatic until rupture, the underestimation of the mortality burden of AA due to inadequate medical conditions in non-developed regions may be the reason for the significant difference in disease burden between regions. However, increases in mortality in low-middle, middle-, and high-middle SDI regions over the past 30 years were still observed in our study, confirming the results of other studies [16,22]. We observed a faster mortality rate increase in these regions over the past 30 years, suggesting that the mortality burden of AA will increase rapidly in developing countries, as shown in our projections. There is an urgent need for further study to clarify the burden of AA in these regions and develop measures to control its increasing trend.

The occurrence of AA has been reported to be associated with smoking, hypertension, atherosclerosis, a high-sodium diet, and age [23–25]. Smoking is considered one of the most critical risk factors for AA, and the decline in smoking prevalence in developed countries over the past 30 years is considered an important reason for the decline in the disease burden of AA [16]. This is also confirmed by the substantial decrease in the proportion of AA deaths attributable to smoking in high- and high-middle SDI regions observed over the past 30 years in our study. However, the current proportion of AA-related deaths attributable to smoking remains high. There has been a rapid increase in AA-related deaths attributable to smoking in recent years. Smoking is associated with AA expansion and rupture [26], and clinicians should strongly advise patients with AA to abstain from tobacco use; moreover, the government should further strengthen tobacco control measures to limit the increased burden of disease at the societal level.

HSBP is associated with both the AA onset and rupture [23,24,26]. Notably, except in high SDI regions, HSBP has not been sufficiently controlled and has been considered an important risk factor for AA in the past two decades. Thus, the accumulated risk attributable to HSBP is continuously increasing, and HSBP will replace smoking as the leading risk factor for AA-related death. We observed that the proportions of HSBP-attributable AA-related deaths in different SDI regions had trends similar to those of the ASMRs in the corresponding regions over the past 30 years, suggesting that HSBP may be able to explain the increasing global burden of AA-related deaths in recent years to some extent. There were sex differences in the contribution of HSBP, with smoking and HSBP being the most important risk factors in men and women, respectively, in 2019. At the same time, we observed that the contributions of smoking and HSBP differed among age groups; there was a tendency for HSBP to become more important with age, which may explain the rapid decline in the sex ratio of AA-related mortality that we observed in individuals aged more than 65 years.

The finding from this analysis suggested that HSBP should be considered the first-degree risk factor in the guidance of AA management. Blood pressure control, even intensive blood pressure control, should be emphasized in patients who have been diagnosed with AA. A most recent meta-analysis has shown that the risk of rupture in AAA patients with comorbid hypertension is 1.66 times higher than that in patients without comorbid hypertension, and the risk of rupture increases by 14% and 28% for every 20 mmHg increase in systolic and 10 mmHg increase in diastolic blood pressure, respectively [27]. For TAA, a large population-based study showed a significant positive correlation between mean systolic blood pressure and TAA mortality trends [28]. However, whether patients with AA need more stringent blood pressure control goals is currently inconclusive. Antihypertensive drugs, particularly angiotensin-converting enzyme inhibitors and angiotensin-receptor blockers, have been suggested to reduce the rate of AA expansion [29,30], but recent studies do not support this conclusion [31]. Given the potential benefits of cardiovascular disease treatment, current guidelines recommend that patients with AA and hypertension seek appropriate treatment, although there is no conclusive evidence to support this [32–35]. Long-term and well-designed clinical studies are desperately needed to keep the optimal blood pressure control target.

In addition, AA screening also should be emphasized in the population of patients with HSBP. Because of the poor prognosis of AA rupture, population-based screening and interventions targeting those at high risk of rupture have been considered possible ways to reduce mortality due to AA. A series of large population-based studies conducted in the 1990s demonstrated that screening in men over 65 reduced AAA-related mortality [36–38]. Based on these findings, the United Kingdom, Sweden, New Zealand, and the USA all conduct AAA screening in men over 65 years of age [39]. However, considering the results of long-term follow-up studies and the observed decline in the prevalence of AAA in recent years, whether AAA screening should be performed has been debated [4,17,40]. Based on our findings, it may be inappropriate to eliminate screening because of the increasing trend in the future mortality burden of AA. Regarding TAA, the benefit of screening the general population is minimal because the disease burden of TAA is much lower than that of AAA. However, screening may be worth considering for patients with a family history of genetic disorders such as Marfan syndrome [34].

More appropriate selection of high-risk populations for AAA screening may yield greater cost-effectiveness. Most current AAA screening methods use >65 years old, and male sex as conditions for screening; the US Preventive Services Task Force Recommendation Statement released in 2019 added smoking as a condition, recommending screening in men aged 65–75 years who smoke rather than the general population aged 65–75 years [41]. However, until now, there has been no screening strategy that emphasizes the role of HSBP [35,41]. Based on our findings, the presence or absence of HSBP should also be used as a criterion to identify subjects who would benefit from screening; otherwise, the effectiveness of screening may be reduced, though this conclusion needs to be confirmed in future studies. We observed a significantly smaller difference between males and females in patients of advanced age. Therefore, further studies are needed to determine whether older women, especially those with combined hypertension who are smokers, should undergo screening.

The treatment of aortic aneurysms has changed dramatically since the introduction of endovascular aortic repair (EVAR) in 1991 [42]. For example, thoracic endovascular aortic aneurysm repair (TEVAR) has replaced open surgery as the primary treatment modality for thoracic aortic aneurysms in the United States. It has significantly reduced the incidence of postoperative adverse events [43,44]. However, aortic aneurysm repair surgery requires a high level of local medical experts, both for open surgery and EVAR. This may lead to significant differences in AA treatment approaches between SDI regions, affecting the burden of AA deaths in the corresponding regions. The rapid decline in AA death burden in high SDI regions from 1990 onwards is demonstrated in our study, which is consistent with the prevalence of endovascular AA repair techniques. However, a similar change was not observed in the low to middle SDI regions. Although EVAR reduced mortality after AA repair in the short term, its long-term prognosis was not superior to open surgery [42]. This result may also be an important reason why the burden of AA death in high SDI regions is no longer decreasing in the recent 5 years.

Despite tremendous advances in AA repair technology, the mortality rate of acute AA rupture has remained high in recent years. The mortality rate once rupture occurs is more than 80%, and even half of the patients with ruptured AA in developed countries die before reaching the hospital [45,46]. One study has shown that despite recent advances in diagnostic techniques and care, this has not reduced mortality in the first 24 h in patients with acute thoracic aortic dissection [47]. This situation demonstrates the importance of having an emergency system with rapid response capability and further illustrates the value of screening for AA in high-risk populations.

### Limitations

The GBD provided data on the burden of AA-related deaths and four associated risk factors from 1990 to 2019. Limitations common to the GBD study have been described in other researches [8,48]. As a global epidemiological study, our study has some inherent limitations determined by the nature of the study. The first and most important limitation is our inability to obtain individual-level data. This limitation prevents our study from analysing the impact of different conditions (e.g. out-of-hospital rupture or elective surgery) or various interventions (e.g. open surgery or EVAR) on mortality. Second, we could not analyse data not provided by the GBD database, such as differences in different subtypes of AA (e.g. TAA or AAA) and the impact of other risk factors (e.g. lipids) on the disease burden of AA. These questions need to be confirmed by future studies. Also, we were unable to obtain data on the burden of death in individuals aged less than 15 years and risk factors in individuals aged less than 30 years, but because the prevalence is extremely low in adolescents and primarily due to genetic factors, we do not believe this affects our conclusions. Third, there may be heterogeneity in the data from different regions due to the great variation in the level of development between different regions of the world. However, GBD, a well-known public health database, has used a mature and well-established model for calibration to minimize the impact of this problem [8].

In addition to the inherent limitations of the study, the characteristics of the AA may have an impact on the accuracy of the study. Among them, the most important reason comes from the hiding of AA and the very high mortality rate in a short time once rupture occurs [45,46]. Because the data sources for GBD are primarily national civil registration and vital statistics, the current background of declining autopsy rates in various countries may result in a proportion of patients who die from ruptured AA not being detected [49]. This bias may be reduced by advances in diagnostic techniques. However, the impact of advances in diagnostic technology on the burden of AA deaths is complex. On the one hand, identifying more patients who die because of acute AA rupture may increase the observed mortality from AA. On the other hand, it may also allow more AA patients to be identified earlier in screening and receive interventions, thus reducing mortality [5,38]. The magnitude of these two effects may vary with the level of social development. Therefore, the impact of advances in diagnostic technology on AA prognosis in terms of AA screening, treatment, and management needs to be discussed in further studies. Furthermore, because of the relatively low prevalence of AA in the population, small-sample studies may introduce bias even if they are based exclusively on autopsy [50]. Therefore, although AA epidemiological studies based on current data sources may underestimate the burden, they are still an important approach systematically to evaluate the global AA burden. More importantly, we do not believe that an underestimated result detracts from the accuracy of the conclusion that this study emphasizes the importance of prevention of the disease.

## Conclusions

Our study found that the death burden of AA is beginning to rebound globally, and this trend will continue for at least the next decade. Additionally, HSBP will surpass smoking as the top risk factor contributing to the death burden of AA in the following decade. Our findings support HSBP being considered as the first-degree risk factor in the guidance of AA management and criteria for population-based screening programs; this will result in a more appropriate screening strategy. For patients diagnosed with AA, establishing a proper standard of blood pressure control as soon as possible may be necessary in reducing the risk of death due to AA.

## Supplementary Material

Supplemental Material

## Data Availability

The data used in this paper are from a public database, which can be accessed by everyone through the links provided in the paper.

## Abbreviation

AA: aortic aneurysm
GBD: Global Burden of Disease Study
BAPC: Bayesian age-period-cohort model
SDI: socio-demographic index
ASMR: age-standardized mortality rate
UI: uncertainty interval
HSBP: high systolic blood pressure
TMREL: theoretical minimum risk exposure level
AAPC: average annual percent change
APC: annual percent change
