# Supplementary material for: Projection of global burden and risk factors for aortic aneurysm – timely warning for greater emphasis on managing blood pressure
Source: Ann Med. 2022 Feb 10;54(1):553–64. doi: 10.1080/07853890.2022.2034932 (PMC8843207; doi:10.1080/07853890.2022.2034932)
Supplement: Supplemental Material [file IANN_A_2034932_SM0233.pdf]

SUPPLEMENTAL MATERIAL

Title: Projection of global burden and risk factors for aortic aneurysm – Timely warning for greater emphasis on managing blood pressure

Content **Page**

OnlineTable	I. Analysis of aortic aneurysm related age-standardized mortality rates from 1990 to 2019 using Joinpoint regression	1
OnlineTable	II. Results of prediction of aortic aneurysm death burden	2
OnlineTable	III.Burden of aortic aneurysm death at the national or regional level	4

Online Table I. Analysis of aortic aneurysm related age-standardized mortality rates from 1990 to 2019 using Joinpoint regression

Location	Trend 1		Trend 2		Trend 3		Trend 4		Trend 5		Trend 6	
	Segment	APC (95%CI)	Segment	APC (95%CI)	Segment	APC (95%CI)	Segment	APC (95%CI)	Segment	APC (95%CI)	Segment	APC (95%CI)
Global	1990 - 1994	0.829 (0.583, 1.076)	1994 - 2000	-0.478 (-0.636, -0.319)	2000 - 2009	-1.675 (-1.747, -1.602)	2009 - 2013	-1.185 (-1.500, -0.869)	2013 - 2017	-0.307 (-0.609, -0.004)	2017 - 2019	0.527 (-0.046, 1.103)
**SDI**												
High SDI	1990 - 1993	0.242 (-0.110, 0.596)	1993 - 1999	-0.952 (-1.102, -0.802)	1999 - 2003	-2.177 (-2.502, -1.851)	2003 - 2009	-3.001 (-3.147, -2.855)	2009 - 2015	-1.565 (-1.713, -1.417)	2015 - 2019	0.637 (0.433, 0.842)
High-middle SDI	1990 - 1994	3.717 (2.980, 4.459)	1994 - 2000	0.499 (0.050, 0.950)	2000 - 2013	-0.830 (-0.936, -0.723)	2013 - 2019	-0.125 (-0.399, 0.149)	/	/	/	/
Middle SDI	1990 - 1997	0.886 (0.765, 1.008)	1997 - 2000	0.317 (-0.481, 1.121)	2000 - 2016	-0.320 (-0.348, -0.292)	2016 - 2019	0.702 (0.425, 0.980)	/	/	/	/
Low-middle SDI	1990 - 2001	0.547 (0.402, 0.693)	2001 - 2004	-1.117 (-2.949, 0.749)	2004 - 2008	0.801 (-0.083, 1.693)	2008 - 2011	-0.733 (-2.343, 0.905)	2011 - 2019	0.970 (0.815, 1.126)	/	/
Low SDI	1990 - 1998	-0.386 (-0.494, -0.278)	1998 - 2005	-1.710 (-1.864, -1.555)	2005 - 2010	-0.352 (-0.627, -0.076)	2010 - 2017	0.113 (-0.019, 0.246)	2017 - 2019	0.886 (0.180, 1.597)	/	/

ASMR, age-standardized mortality rate; APC, annual percent change; SDI, socio-demographic index; CI, confidence interval.

Online Table II. Results of prediction of aortic aneurysm death burden.

Sex	Location	2030 ASMR (95%CI)	2030 Case (95%CI)	2020-2030 AAPC (95%CI)	*P* value
**Both**					
	Global	2.238(1.762, 2.715)	244685(227177, 262193)	0.318(0.288, 0.348)	< 0.001
	**SDI**				
	High SDI	3.134(2.264, 4.005)	84166(75656, 92677)	0.481(0.429, 0.534)	< 0.001
	High-middle SDI	2.182(1.427, 2.936)	59631(52640, 66622)	-0.049(-0.087, -0.010)	0.013
	Middle SDI	1.550(1.220, 1.879)	48949(45436, 52461)	0.544(0.537, 0.550)	< 0.001
	Low-middle SDI	1.918(1.439, 2.398)	33873(31053, 36693)	1.183(1.166, 1.200)	< 0.001
	Low SDI	1.959(1.426, 2.492)	12602(11465, 13740)	0.565(0.554, 0.576)	< 0.001
	**Risk**				
	All risk factors	1.229(0.942, 1.516)	135836(125011, 146662)	0.301(0.265, 0.337)	< 0.001
	High systolic blood pressure	0.792(0.599, 0.985)	87606(80365, 94847)	0.657(0.613, 0.701)	< 0.001
	Smoking	0.715(0.543, 0.887)	79578(72854, 86301)	-0.074(-0.107, -0.040)	< 0.001
	Diet high in sodium	0.115(0.087, 0.144)	12878(11752, 14005)	0.321(0.299, 0.343)	< 0.001
	Lead exposure	0.038(0.027, 0.049)	4284(3825, 4742)	-0.943(-0.950, -0.935)	< 0.001
**Male**					
	Global	3.212(2.464, 3.960)	157682(145198, 170166)	0.415(0.375, 0.455)	< 0.001
	**SDI**				
	High SDI	4.482(3.084, 5.880)	52353(46383, 58323)	0.785(0.715, 0.854)	< 0.001
	High-middle SDI	3.311(2.017, 4.604)	39438(34109, 44768)	-0.228(-0.290, -0.167)	< 0.001
	Middle SDI	2.267(1.756, 2.778)	32578(30068, 35088)	0.640(0.630, 0.651)	< 0.001
	Low-middle SDI	2.664(1.961, 3.366)	21938(19984, 23892)	1.262(1.244, 1.281)	< 0.001
	Low SDI	2.486(1.817, 3.155)	7882(7162, 8602)	0.352(0.341, 0.362)	< 0.001
	**Risk**				
	All risk factors	1.908(1.441, 2.374)	97161(88879, 105442)	0.256(0.214, 0.298)	< 0.001

	High systolic blood pressure	1.126(0.836, 1.415)	57200(52072, 62328)	0.673(0.623, 0.724)	< 0.001
	Smoking	1.269(0.955, 1.584)	65873(60108, 71638)	-0.130(-0.169, -0.090)	< 0.001
	Diet high in sodium	0.184(0.135, 0.232)	9474(8566, 10382)	0.250(0.223, 0.278)	< 0.001
	Lead exposure	0.062(0.043, 0.081)	3110(2749, 3471)	-1.102(-1.103, -1.101)	< 0.001
**Female**					
	Global	1.495(1.188, 1.801)	92035(85447, 98622)	0.339(0.320, 0.359)	< 0.001
	**SDI**				
	High SDI	2.262(1.700, 2.825)	37427(33892, 40961)	0.638(0.607, 0.669)	< 0.001
	High-middle SDI	1.296(0.921, 1.671)	20602(18504, 22701)	0.098(0.093, 0.104)	< 0.001
	Middle SDI	0.945(0.718, 1.172)	16140(14780, 17500)	0.327(0.324, 0.329)	< 0.001
	Low-middle SDI	1.279(0.958, 1.600)	11972(10926, 13018)	1.156(1.142, 1.171)	< 0.001
	Low SDI	1.457(0.962, 1.953)	4674(4130, 5218)	0.905(0.888, 0.921)	< 0.001
	**Risk**				
	All risk factors	0.655(0.491, 0.818)	39723(36326, 43121)	0.384(0.359, 0.409)	< 0.001
	High systolic blood pressure	0.510(0.373, 0.646)	31182(28327, 34038)	0.633(0.601, 0.666)	< 0.001
	Smoking	0.232(0.166, 0.298)	13815(12414, 15216)	-0.152(-0.171, -0.134)	< 0.001
	Diet high in sodium	0.055(0.039, 0.071)	3366(3009, 3724)	0.257(0.246, 0.268)	< 0.001
	Lead exposure	0.019(0.012, 0.025)	1156(997, 1315)	-0.716(-0.726, -0.705)	< 0.001

AAPC, average annual percent change; SDI, socio-demographic index; CI, confidence interval.

Online Table Ⅲ. Burden of aortic aneurysm death at the national or regional level

Location	SDI	2019 ASMR (95%UI）	2019 Case (95%UI）	1990-2019 AAPC (95%CI）	2030 ASMR (95%CI）	2030 Case (95%CI）	2020-2030 AAPC (95%CI）
Afghanistan	Low SDI	0.998 (0.638, 1.582)	109 (66, 181)	-0.143 (-0.178, -0.107)	0.809 (0.401, 1.217)	161 (123, 199)	-0.876 (-0.942, -0.809)
Albania	Middle SDI	1.148 (0.875, 1.507)	48 (37, 63)	-0.128 (-0.751, 0.499)	0.921 (0.380, 1.462)	55 (35, 76)	-0.948 (-0.967, -0.929)
Algeria	Middle SDI	1.159 (0.876, 1.533)	318 (235, 431)	-0.554 (-0.625, -0.482)	0.934 (0.566, 1.303)	469 (389, 548)	-0.090 (-0.115, -0.066)
American Samoa	High-middle SDI	2.514 (2.125, 2.939)	1 (1, 1)	-1.997 (-2.495, -1.497)	/	/	/
Andorra	High SDI	5.543 (3.881, 7.633)	8 (6, 11)	-0.626 (-0.709, -0.543)	/	/	/
Angola	Low-middle SDI	3.006 (2.290, 3.773)	271 (202, 348)	-0.548 (-0.740, -0.357)	3.364 (1.787, 4.941)	468 (379, 557)	1.565 (1.550, 1.580)
Antigua and Barbuda	High-middle SDI	2.560 (2.149, 2.973)	2 (2, 3)	-0.205 (-1.006, 0.603)	/	/	/
Argentina	High-middle SDI	3.470 (3.123, 3.811)	1909 (1716, 2105)	-0.615 (-0.967, -0.262)	4.256 (1.827, 6.686)	3027 (2399, 3655)	2.344 (2.287, 2.401)
Armenia	Middle SDI	8.745 (7.337, 10.290)	356 (299, 422)	2.180 (1.787, 2.575)	10.020 (4.390, 15.650)	500 (381, 620)	1.351 (1.335, 1.367)
Australia	High SDI	2.941 (2.537, 3.264)	1354 (1160, 1509)	-3.072 (-3.287, -2.857)	3.754 (1.400, 6.108)	2488 (1858, 3118)	3.185 (3.079, 3.291)
Austria	High SDI	2.215 (1.989, 2.440)	420 (373, 467)	-1.189 (-1.303, -1.074)	2.046 (1.008, 3.084)	504 (396, 611)	-0.105 (-0.132, -0.078)
Azerbaijan	Middle SDI	0.920	65	1.177	0.755	87	-0.475

		(0.782, 1.072)	(54, 78)	(0.991, 1.363)	(0.323, 1.186)	(61, 114)	(-0.481, -0.469)
Bahamas	High-middle SDI	3.874<br>(3.148, 4.756)	14<br>(11, 17)	0.080<br>(-0.318, 0.479)	/	/	/
Bahrain	High-middle SDI	1.460<br>(1.139, 1.833)	9<br>(7, 12)	-0.384<br>(-0.865, 0.099)	/	/	/
Bangladesh	Low-middle SDI	1.642<br>(1.173, 2.173)	1878<br>(1346, 2497)	0.496<br>(0.139, 0.854)	2.943<br>(1.293, 4.593)	4407<br>(3512, 5302)	4.662<br>(4.650, 4.675)
Barbados	High-middle SDI	2.761<br>(2.256, 3.303)	14<br>(11, 16)	0.295<br>(-0.251, 0.843)	/	/	/
Belarus	High-middle SDI	2.395<br>(1.855, 3.110)	382<br>(295, 491)	1.682<br>(1.190, 2.175)	2.462<br>(1.460, 3.464)	452<br>(372, 532)	0.476<br>(0.466, 0.486)
Belgium	High SDI	2.794<br>(2.494, 3.080)	735<br>(645, 818)	-1.524<br>(-1.943, -1.103)	2.841<br>(1.326, 4.356)	935<br>(722, 1147)	0.900<br>(0.853, 0.947)
Belize	Low-middle SDI	1.684<br>(1.402, 1.980)	4<br>(3, 5)	0.577<br>(-0.056, 1.213)	/	/	/
Benin	Low SDI	1.548<br>(1.113, 2.003)	66<br>(48, 86)	-0.885<br>(-1.099, -0.670)	1.384<br>(0.637, 2.130)	99<br>(71, 126)	0.156<br>(0.102, 0.210)
Bermuda	High SDI	5.701<br>(4.705, 6.936)	8<br>(7, 10)	-1.456<br>(-1.662, -1.249)	/	/	/
Bhutan	Low-middle SDI	2.146<br>(1.457, 2.896)	11<br>(7, 14)	1.344<br>(1.279, 1.409)	/	/	/
Bolivia (Plurinational State of)	Low-middle SDI	2.213<br>(1.515, 2.910)	170<br>(115, 226)	0.558<br>(0.479, 0.637)	1.816<br>(0.964, 2.668)	253<br>(201, 306)	0.430<br>(0.406, 0.454)
Bosnia and Herzegovina	High-middle SDI	3.756<br>(3.040, 4.597)	212<br>(170, 259)	1.033<br>(0.269, 1.803)	3.810<br>(2.116, 5.503)	283<br>(224, 341)	0.407<br>(0.404, 0.411)
Botswana	Middle SDI	2.381	27	-0.714	1.662	34	-1.293

		(1.766, 3.175)	(19, 36)	(-0.886, -0.542)	(0.445, 2.878)	(19, 50)	(-1.356, -1.231)
Brazil	Middle SDI	4.577<br>(4.183, 4.892)	10533<br>(9670, 11249)	1.000<br>(0.825, 1.175)	4.708<br>(3.382, 6.035)	15485<br>(14021, 16949)	0.074<br>(0.065, 0.083)
Brunei Darussalam	High SDI	6.81<br>(5.954, 7.720)	13<br>(12, 15)	0.14<br>(-0.330, 0.612)	/	/	/
Bulgaria	High-middle SDI	2.725<br>(2.142, 3.401)	364<br>(287, 455)	1.678<br>(1.294, 2.065)	2.840<br>(1.525, 4.155)	400<br>(323, 477)	0.843<br>(0.827, 0.860)
Burkina Faso	Low SDI	1.825<br>(1.160, 2.611)	142<br>(89, 205)	-0.079<br>(-0.236, 0.079)	2.941<br>(1.574, 4.307)	226<br>(176, 275)	0.517<br>(0.502, 0.532)
Burundi	Low SDI	2.025<br>(1.156, 2.895)	76<br>(43, 112)	-2.042<br>(-2.170, -1.913)	1.916<br>(0.715, 3.117)	103<br>(71, 134)	-0.833<br>(-0.874, -0.791)
Cabo Verde	Low-middle SDI	2.027<br>(1.744, 2.355)	8<br>(7, 10)	2.527<br>(1.719, 3.341)	/	/	/
Cambodia	Low-middle SDI	1.262<br>(0.975, 1.706)	124<br>(95, 167)	0.751<br>(0.707, 0.795)	1.868<br>(0.961, 2.776)	217<br>(170, 265)	1.183<br>(1.154, 1.211)
Cameroon	Low-middle SDI	1.714<br>(1.226, 2.452)	175<br>(122, 254)	-0.600<br>(-0.770, -0.429)	2.472<br>(1.352, 3.593)	282<br>(227, 338)	1.08<br>(1.062, 1.099)
Canada	High SDI	2.736<br>(2.387, 3.054)	2031<br>(1764, 2273)	-2.790<br>(-2.921, -2.660)	3.821<br>(1.375, 6.267)	4077<br>(3062, 5092)	3.846<br>(3.740, 3.952)
Central African Republic	Low SDI	2.786<br>(1.718, 3.989)	50<br>(30, 76)	-1.196<br>(-1.370, -1.021)	3.022<br>(1.232, 4.812)	77<br>(52, 102)	1.792<br>(1.732, 1.852)
Chad	Low SDI	1.471<br>(0.943, 2.096)	71<br>(45, 104)	-0.504<br>(-0.625, -0.384)	1.510<br>(0.704, 2.316)	108<br>(78, 137)	0.306<br>(0.246, 0.366)
Chile	High-middle SDI	2.668<br>(2.369, 2.939)	637<br>(565, 702)	0.501<br>(0.240, 0.763)	2.504<br>(1.378, 3.630)	902<br>(745, 1060)	0.372<br>(0.336, 0.407)
China	Middle SDI	0.931	17038	-0.262	1.027	25498	0.342

		(0.787, 1.085)	(14392, 19980)	(-0.372, -0.152)	(0.740, 1.315)	(22934, 28061)	(0.325, 0.360)
Colombia	Middle SDI	2.849<br>(2.181, 3.656)	1515<br>(1159, 1939)	-0.568<br>(-1.187, 0.054)	3.797<br>(1.073, 6.521)	3023<br>(2235, 3810)	2.619<br>(2.549, 2.688)
Comoros	Low-middle SDI	2.248<br>(1.645, 2.908)	10<br>(7, 13)	-1.351<br>(-1.677, -1.024)	/	/	/
Congo	Low-middle SDI	3.342<br>(2.652, 4.141)	71<br>(54, 90)	-1.176<br>(-1.453, -0.898)	4.205<br>(1.653, 6.757)	112<br>(79, 146)	0.305<br>(0.273, 0.337)
Cook Islands	High-middle SDI	3.224<br>(2.597, 3.978)	1<br>(1, 1)	-1.043<br>(-1.088, -0.998)	/	/	/
Costa Rica	Middle SDI	2.683<br>(2.046, 3.468)	136<br>(103, 174)	1.189<br>(0.552, 1.830)	2.473<br>(1.254, 3.692)	221<br>(172, 270)	0.650<br>(0.602, 0.699)
Croatia	High-middle SDI	3.816<br>(3.003, 4.713)	344<br>(272, 424)	2.289<br>(2.088, 2.491)	3.666<br>(1.969, 5.362)	374<br>(297, 451)	-0.077<br>(-0.083, -0.071)
Cuba	Middle SDI	4.019<br>(3.243, 4.952)	796<br>(642, 980)	-0.315<br>(-0.677, 0.047)	4.350<br>(1.930, 6.771)	1205<br>(923, 1487)	1.233<br>(1.209, 1.256)
Cyprus	High SDI	4.066<br>(3.495, 4.729)	78<br>(66, 90)	-1.789<br>(-2.144, -1.433)	3.482<br>(1.423, 5.541)	92<br>(61, 122)	-0.487<br>(-0.541, -0.433)
Czechia	High SDI	2.856<br>(2.338, 3.452)	613<br>(501, 741)	0.983<br>(0.640, 1.327)	2.456<br>(1.428, 3.484)	654<br>(537, 770)	-1.143<br>(-1.159, -1.127)
Côte d'Ivoire	Low SDI	1.641<br>(1.218, 2.141)	142<br>(102, 190)	-0.916<br>(-1.168, -0.664)	/	/	/
Democratic People's Republic of Korea	Low-middle SDI	1.229<br>(1.033, 1.481)	378<br>(314, 462)	0.006<br>(-0.029, 0.040)	1.305<br>(0.804, 1.807)	488<br>(409, 566)	0.094<br>(0.064, 0.125)
Democratic Republic of the Congo	Low SDI	2.558<br>(1.806, 3.385)	759<br>(539, 1015)	-1.046<br>(-1.280, -0.811)	2.650<br>(1.600, 3.700)	1245<br>(1058, 1432)	0.838<br>(0.786, 0.890)
Denmark	High SDI	5.323	671	-0.610	5.120	857	0.279

		(4.721, 5.956)	(592, 752)	(-0.748, -0.473)	(2.599, 7.641)	(669, 1045)	(0.245, 0.312)
Djibouti	Low-middle SDI	2.346<br>(1.654, 3.239)	11<br>(7, 16)	-1.001<br>(-1.137, -0.865)	/	/	/
Dominica	High-middle SDI	4.915<br>(4.068, 5.924)	5<br>(4, 5)	-0.066<br>(-0.165, 0.033)	/	/	/
Dominican Republic	Low-middle SDI	2.073<br>(1.612, 2.640)	183<br>(141, 234)	1.501<br>(1.110, 1.893)	2.167<br>(1.165, 3.170)	297<br>(239, 355)	0.791<br>(0.763, 0.818)
Ecuador	Middle SDI	2.002<br>(1.593, 2.539)	276<br>(218, 351)	0.255<br>(-0.083, 0.594)	1.449<br>(0.419, 2.480)	342<br>(246, 437)	-1.775<br>(-1.830, -1.720)
Egypt	Middle SDI	1.440<br>(1.034, 2.083)	757<br>(536, 1098)	0.657<br>(0.153, 1.164)	1.338<br>(0.858, 1.818)	1164<br>(1006, 1322)	1.022<br>(1.018, 1.025)
El Salvador	Low-middle SDI	0.920<br>(0.699, 1.175)	57<br>(43, 73)	0.168<br>(-0.318, 0.656)	0.919<br>(0.398, 1.440)	77<br>(53, 101)	0.348<br>(0.306, 0.390)
Equatorial Guinea	Middle SDI	3.021<br>(2.169, 4.313)	12<br>(8, 17)	-0.416<br>(-0.576, -0.256)	/	/	/
Eritrea	Low SDI	2.528<br>(1.701, 3.557)	55<br>(36, 80)	-0.800<br>(-0.983, -0.616)	3.017<br>(1.097, 4.936)	76<br>(52, 100)	1.216<br>(1.163, 1.270)
Estonia	High SDI	2.894<br>(2.209, 3.816)	79<br>(60, 104)	0.447<br>(0.032, 0.864)	2.121<br>(0.883, 3.359)	72<br>(48, 95)	-1.775<br>(-1.782, -1.768)
Eswatini	Low-middle SDI	2.232<br>(1.693, 2.973)	11<br>(8, 15)	-0.684<br>(-0.836, -0.532)	/	/	/
Ethiopia	Low SDI	1.695<br>(1.079, 2.224)	597<br>(387, 786)	-1.668<br>(-1.847, -1.489)	1.400<br>(0.665, 2.135)	913<br>(742, 1084)	0.506<br>(0.481, 0.532)
Fiji	Middle SDI	5.398<br>(4.357, 6.556)	31<br>(24, 39)	-0.154<br>(-0.407, 0.100)	6.349<br>(2.301, 10.398)	46<br>(29, 64)	0.709<br>(0.668, 0.750)
Finland	High SDI	4.838	654	-1.194	4.272	737	-0.752

		(4.299, 5.408)	(575, 736)	(-1.328, -1.060)	(2.479, 6.064)	(594, 881)	(-0.774, -0.729)
France	High SDI	2.144<br>(1.901, 2.378)	3401<br>(2956, 3809)	-1.578<br>(-1.739, -1.417)	2.756<br>(1.280, 4.233)	5314<br>(4218, 6409)	2.654<br>(2.592, 2.716)
Gabon	Middle SDI	3.711<br>(2.926, 4.621)	34<br>(26, 43)	-0.692<br>(-0.823, -0.560)	3.571<br>(1.332, 5.810)	46<br>(28, 64)	0.108<br>(0.094, 0.122)
Gambia	Low SDI	1.719<br>(1.330, 2.171)	15<br>(11, 19)	-0.179<br>(-0.833, 0.479)	/	/	/
Georgia	High-middle SDI	2.303<br>(1.865, 2.736)	139<br>(112, 166)	3.775<br>(3.233, 4.319)	2.109<br>(-0.591, 4.809)	180<br>(81, 278)	1.512<br>(1.293, 1.732)
Germany	High SDI	2.218<br>(1.999, 2.427)	4558<br>(4040, 5041)	-1.151<br>(-1.312, -0.989)	2.337<br>(1.500, 3.175)	5434<br>(4705, 6163)	0.770<br>(0.728, 0.813)
Ghana	Low-middle SDI	1.429<br>(1.136, 1.812)	196<br>(151, 251)	-1.493<br>(-1.703, -1.282)	2.345<br>(-1.141, 5.830)	439<br>(124, 755)	3.914<br>(3.592, 4.237)
Greece	High-middle SDI	4.399<br>(3.907, 4.848)	1105<br>(970, 1223)	0.876<br>(0.722, 1.031)	4.664<br>(2.734, 6.594)	1366<br>(1148, 1584)	0.936<br>(0.913, 0.960)
Greenland	High-middle SDI	2.254<br>(1.844, 2.692)	1<br>(1, 2)	-1.493<br>(-2.135, -0.846)	/	/	/
Grenada	Middle SDI	4.650<br>(4.046, 5.257)	5<br>(4, 5)	-0.095<br>(-0.871, 0.687)	/	/	/
Guam	High SDI	2.338<br>(1.940, 2.782)	4<br>(4, 5)	-3.761<br>(-4.550, -2.966)	/	/	/
Guatemala	Low-middle SDI	1.044<br>(0.842, 1.298)	107<br>(85, 134)	-0.229<br>(-0.772, 0.317)	0.958<br>(0.451, 1.465)	152<br>(114, 190)	-0.087<br>(-0.098, -0.076)
Guinea	Low SDI	1.695<br>(1.203, 2.358)	84<br>(59, 119)	-0.425<br>(-0.519, -0.332)	2.070<br>(0.989, 3.151)	112<br>(82, 142)	-1.032<br>(-1.093, -0.970)
Guinea-Bissau	Low SDI	1.839	11	-0.796	/	/	/

		(1.342, 2.504)	(8, 16)	(-0.876, -0.716)			
Guyana	Middle SDI	4.517<br>(3.542, 5.575)	25<br>(19, 31)	1.695<br>(1.262, 2.131)	3.922<br>(0.871, 6.973)	39<br>(22, 57)	0.992<br>(0.910, 1.075)
Haiti	Low SDI	3.286<br>(2.046, 4.838)	187<br>(114, 283)	-0.418<br>(-0.505, -0.331)	2.901<br>(1.642, 4.161)	272<br>(218, 325)	0.336<br>(0.291, 0.381)
Honduras	Low-middle SDI	1.471<br>(1.142, 1.901)	76<br>(59, 98)	1.427<br>(1.192, 1.662)	1.525<br>(0.683, 2.367)	135<br>(98, 171)	0.539<br>(0.515, 0.562)
Hungary	High-middle SDI	2.860<br>(2.355, 3.425)	562<br>(462, 672)	0.180<br>(-0.205, 0.567)	2.576<br>(1.530, 3.622)	600<br>(494, 706)	-0.867<br>(-0.868, -0.865)
Iceland	High SDI	3.015<br>(2.581, 3.450)	18<br>(15, 21)	-1.088<br>(-1.299, -0.877)	/	/	/
India	Low-middle SDI	1.678<br>(1.302, 2.044)	16765<br>(12739, 20687)	0.113<br>(-0.140, 0.366)	2.058<br>(1.361, 2.755)	28942<br>(25404, 32481)	1.715<br>(1.682, 1.748)
Indonesia	Middle SDI	1.670<br>(1.226, 2.322)	2773<br>(2013, 3880)	1.262<br>(1.210, 1.314)	1.577<br>(1.110, 2.044)	4073<br>(3624, 4523)	-0.177<br>(-0.180, -0.174)
Iran (Islamic Republic of)	Middle SDI	1.017<br>(0.901, 1.122)	671<br>(598, 740)	-0.298<br>(-0.465, -0.131)	1.108<br>(0.664, 1.553)	974<br>(824, 1125)	-0.141<br>(-0.173, -0.109)
Iraq	Middle SDI	0.871<br>(0.708, 1.040)	170<br>(134, 210)	0.334<br>(0.214, 0.455)	1.162<br>(0.634, 1.689)	245<br>(195, 295)	0.938<br>(0.927, 0.948)
Ireland	High SDI	4.241<br>(3.692, 4.791)	331<br>(287, 374)	-0.945<br>(-1.210, -0.680)	4.181<br>(1.484, 6.877)	487<br>(346, 628)	0.362<br>(0.285, 0.438)
Israel	High-middle SDI	1.664<br>(1.467, 1.842)	204<br>(179, 226)	-1.047<br>(-1.190, -0.902)	1.820<br>(0.823, 2.816)	304<br>(227, 381)	1.052<br>(1.011, 1.093)
Italy	High-middle SDI	2.490<br>(2.247, 2.667)	4032<br>(3548, 4329)	-0.475<br>(-0.619, -0.331)	2.721<br>(1.504, 3.938)	5251<br>(4355, 6147)	1.259<br>(1.217, 1.302)
Jamaica	Middle SDI	2.280	70	1.620	2.439	97	1.114

		(1.817, 2.790)	(56, 86)	(0.922, 2.324)	(1.070, 3.807)	(69, 125)	(1.079, 1.148)
Japan	High SDI	4.513<br>(3.835, 4.907)	20169<br>(16271, 22322)	1.619<br>(1.487, 1.751)	4.622<br>(3.232, 6.012)	23374<br>(20615, 26134)	0.114<br>(0.057, 0.171)
Jordan	High-middle SDI	1.305<br>(1.059, 1.621)	75<br>(61, 95)	-0.990<br>(-1.291, -0.687)	1.591<br>(0.457, 2.725)	122<br>(81, 163)	2.259<br>(2.212, 2.305)
Kazakhstan	High-middle SDI	2.305<br>(1.958, 2.695)	391<br>(331, 460)	1.548<br>(1.040, 2.059)	2.294<br>(1.073, 3.514)	574<br>(450, 698)	0.904<br>(0.882, 0.926)
Kenya	Low-middle SDI	2.280<br>(1.864, 2.740)	407<br>(340, 496)	-0.009<br>(-0.084, 0.065)	2.960<br>(1.753, 4.167)	609<br>(512, 706)	-0.983<br>(-0.995, -0.971)
Kiribati	Low-middle SDI	1.373<br>(1.140, 1.651)	1<br>(1, 1)	-0.402<br>(-0.481, -0.323)	/	/	/
Kuwait	High SDI	1.433<br>(1.111, 1.772)	30<br>(24, 37)	0.716<br>(-0.035, 1.473)	/	/	/
Kyrgyzstan	Low-middle SDI	0.934<br>(0.803, 1.073)	38<br>(32, 44)	0.853<br>(0.381, 1.328)	0.867<br>(0.321, 1.412)	44<br>(27, 61)	-0.705<br>(-0.715, -0.694)
Lao People's Democratic Republic	Low-middle SDI	1.538<br>(1.199, 1.918)	54<br>(41, 68)	0.792<br>(0.733, 0.851)	1.807<br>(0.767, 2.847)	91<br>(64, 119)	1.367<br>(1.322, 1.413)
Latvia	High SDI	2.156<br>(1.784, 2.616)	89<br>(74, 108)	0.164<br>(-0.508, 0.841)	1.538<br>(0.590, 2.487)	73<br>(48, 99)	-1.843<br>(-1.850, -1.837)
Lebanon	High-middle SDI	1.373<br>(1.025, 1.699)	71<br>(53, 88)	-0.291<br>(-0.345, -0.236)	0.962<br>(0.424, 1.500)	75<br>(52, 98)	-2.074<br>(-2.129, -2.019)
Lesotho	Low-middle SDI	2.329<br>(1.685, 3.151)	25<br>(17, 34)	0.057<br>(-0.168, 0.284)	1.997<br>(0.576, 3.418)	34<br>(19, 49)	1.098<br>(0.998, 1.197)
Liberia	Low SDI	1.426<br>(0.999, 1.968)	24<br>(17, 34)	-0.997<br>(-1.148, -0.847)	1.629<br>(0.276, 2.982)	56<br>(30, 81)	1.625<br>(1.340, 1.911)
Libya	High-middle SDI	0.897	42	0.487	1.418	82	2.210

		(0.566, 1.362)	(26, 63)	(0.289, 0.685)	(0.502, 2.335)	(57, 108)	(2.138, 2.282)
Lithuania	High SDI	2.726<br>(2.201, 3.299)	156<br>(127, 189)	1.357<br>(0.386, 2.337)	2.410<br>(1.158, 3.662)	158<br>(119, 196)	-0.749<br>(-0.757, -0.742)
Luxembourg	High SDI	2.613<br>(2.229, 3.070)	28<br>(24, 33)	-1.411<br>(-1.618, -1.204)	/	/	/
Madagascar	Low SDI	3.096<br>(2.167, 4.245)	292<br>(200, 407)	-1.094<br>(-1.253, -0.936)	2.538<br>(1.303, 3.774)	448<br>(364, 532)	0.097<br>(0.057, 0.138)
Malawi	Low SDI	2.312<br>(1.535, 3.052)	146<br>(96, 196)	-0.617<br>(-0.789, -0.445)	2.873<br>(1.519, 4.227)	225<br>(178, 273)	0.447<br>(0.429, 0.465)
Malaysia	High-middle SDI	4.398<br>(3.508, 5.452)	971<br>(770, 1213)	0.265<br>(-0.751, 1.292)	4.542<br>(2.052, 7.032)	1758<br>(1356, 2160)	1.109<br>(1.055, 1.163)
Maldives	Low-middle SDI	2.336<br>(1.936, 2.821)	6<br>(5, 7)	-0.583<br>(-0.763, -0.402)	/	/	/
Mali	Low SDI	1.480<br>(0.999, 2.071)	105<br>(70, 149)	-0.707<br>(-0.793, -0.622)	2.642<br>(1.222, 4.062)	159<br>(119, 199)	1.100<br>(1.091, 1.108)
Malta	High-middle SDI	1.759<br>(1.485, 2.066)	17<br>(15, 20)	-0.984<br>(-1.126, -0.841)	/	/	/
Marshall Islands	Low-middle SDI	4.194<br>(3.031, 5.678)	1<br>(1, 2)	-0.499<br>(-0.581, -0.416)	/	/	/
Mauritania	Low-middle SDI	1.446<br>(1.132, 1.866)	26<br>(20, 35)	-1.540<br>(-1.790, -1.290)	1.839<br>(0.395, 3.283)	48<br>(27, 70)	1.133<br>(1.021, 1.245)
Mauritius	High-middle SDI	1.074<br>(0.861, 1.298)	17<br>(14, 21)	-0.233<br>(-0.810, 0.348)	/	/	/
Mexico	Middle SDI	0.999<br>(0.831, 1.165)	1099<br>(912, 1286)	-0.102<br>(-0.595, 0.393)	1.142<br>(0.724, 1.560)	1809<br>(1558, 2060)	1.494<br>(1.486, 1.502)
Micronesia (Federated States	Low-middle SDI	4.457	3	-0.462	/	/	/

of)		(3.055, 6.102)	(2, 4)	(-0.502, -0.422)			
Monaco	High SDI	4.335<br>(3.435, 5.144)	5<br>(4, 6)	-0.524<br>(-0.577, -0.471)	/	/	/
Mongolia	Low-middle SDI	1.181<br>(0.967, 1.445)	22<br>(17, 28)	0.107<br>(-0.055, 0.269)	/	/	/
Montenegro	High-middle SDI	8.261<br>(6.786, 10.152)	79<br>(65, 97)	0.862<br>(0.563, 1.163)	6.845<br>(2.937, 10.753)	87<br>(60, 113)	-0.960<br>(-0.970, -0.951)
Morocco	Low-middle SDI	1.286<br>(0.949, 1.621)	338<br>(244, 435)	0.711<br>(0.388, 1.034)	1.401<br>(0.824, 1.978)	552<br>(457, 647)	0.507<br>(0.477, 0.537)
Mozambique	Low SDI	2.917<br>(1.761, 4.222)	272<br>(162, 404)	0.091<br>(-0.047, 0.229)	3.077<br>(1.716, 4.437)	426<br>(352, 500)	0.536<br>(0.488, 0.584)
Myanmar	Low-middle SDI	1.441<br>(1.166, 1.756)	566<br>(454, 699)	0.497<br>(0.454, 0.540)	1.818<br>(1.177, 2.458)	811<br>(694, 929)	0.324<br>(0.311, 0.337)
Namibia	Middle SDI	2.620<br>(2.014, 3.321)	32<br>(24, 42)	-0.511<br>(-0.628, -0.394)	2.851<br>(0.361, 5.342)	42<br>(19, 65)	1.056<br>(0.946, 1.166)
Nauru	Middle SDI	4.509<br>(3.232, 5.869)	0<br>(0, 0)	-0.730<br>(-0.819, -0.642)	/	/	/
Nepal	Low SDI	1.717<br>(1.100, 2.320)	326<br>(206, 446)	1.336<br>(1.235, 1.438)	2.715<br>(1.441, 3.988)	654<br>(533, 775)	2.912<br>(2.895, 2.928)
Netherlands	High SDI	4.340<br>(3.827, 4.815)	1612<br>(1414, 1792)	-1.704<br>(-1.800, -1.608)	5.025<br>(1.715, 8.335)	2616<br>(1900, 3331)	2.208<br>(2.121, 2.296)
New Zealand	High SDI	5.317<br>(4.699, 5.919)	448<br>(393, 500)	-2.111<br>(-2.384, -1.837)	5.793<br>(2.198, 9.388)	736<br>(536, 936)	1.757<br>(1.684, 1.831)
Nicaragua	Low-middle SDI	1.099<br>(0.905, 1.297)	41<br>(34, 49)	0.956<br>(0.182, 1.736)	0.971<br>(0.341, 1.600)	67<br>(44, 91)	0.583<br>(0.531, 0.634)
Niger	Low SDI	1.276	81	-1.055	1.254	119	-0.164

		(0.689, 1.881)	(43, 124)	(-1.200, -0.911)	(0.586, 1.923)	(87, 151)	(-0.213, -0.114)
Nigeria	Low-middle SDI	1.600<br>(1.222, 2.087)	1149<br>(868, 1520)	-1.273<br>(-1.401, -1.145)	2.827<br>(1.456, 4.197)	1495<br>(1239, 1751)	-0.877<br>(-0.904, -0.850)
Niue	High-middle SDI	3.273<br>(2.514, 4.124)	0<br>(0, 0)	-0.818<br>(-0.872, -0.764)	/	/	/
North Macedonia	High-middle SDI	2.476<br>(1.968, 3.062)	72<br>(56, 90)	1.719<br>(1.445, 1.994)	2.318<br>(0.929, 3.707)	94<br>(64, 123)	0.298<br>(0.286, 0.310)
Northern Mariana Islands	High-middle SDI	2.287<br>(1.960, 2.627)	1<br>(1, 1)	-4.030<br>(-4.091, -3.969)	/	/	/
Norway	High SDI	5.210<br>(4.693, 5.604)	552<br>(491, 594)	-1.165<br>(-1.344, -0.986)	4.298<br>(2.216, 6.380)	641<br>(498, 784)	-1.054<br>(-1.067, -1.041)
Oman	High-middle SDI	2.246<br>(1.549, 3.032)	29<br>(20, 39)	0.453<br>(0.111, 0.796)	1.054<br>(0.252, 1.856)	40<br>(24, 55)	-3.100<br>(-3.135, -3.065)
Pakistan	Low SDI	2.012<br>(1.520, 2.590)	1835<br>(1354, 2405)	0.865<br>(0.746, 0.984)	1.745<br>(1.171, 2.320)	2816<br>(2496, 3136)	0.763<br>(0.734, 0.792)
Palau	High-middle SDI	2.470<br>(1.808, 3.278)	0<br>(0, 1)	-0.470<br>(-0.513, -0.427)	/	/	/
Palestine	Low-middle SDI	1.075<br>(0.912, 1.251)	21<br>(18, 25)	0.211<br>(-0.097, 0.520)	1.241<br>(0.322, 2.160)	42<br>(24, 61)	1.609<br>(1.490, 1.728)
Panama	Middle SDI	1.862<br>(1.425, 2.370)	78<br>(60, 99)	-0.483<br>(-0.825, -0.139)	1.671<br>(0.555, 2.787)	112<br>(76, 148)	0.220<br>(0.151, 0.289)
Papua New Guinea	Low SDI	2.618<br>(1.621, 4.167)	97<br>(58, 160)	-0.220<br>(-0.312, -0.128)	3.127<br>(1.553, 4.702)	150<br>(114, 186)	0.663<br>(0.615, 0.712)
Paraguay	Middle SDI	2.656<br>(2.029, 3.423)	143<br>(109, 184)	1.148<br>(0.645, 1.653)	2.714<br>(1.218, 4.211)	210<br>(160, 259)	0.432<br>(0.381, 0.483)
Peru	Middle SDI	1.037	335	-0.716	0.928	423	-1.807

		(0.770, 1.349)	(248, 437)	(-1.414, -0.014)	(0.534, 1.323)	(351, 496)	(-1.832, -1.781)
Philippines	Middle SDI	1.686<br>(1.380, 2.014)	1119<br>(903, 1349)	1.750<br>(1.271, 2.231)	1.547<br>(0.158, 2.936)	1779<br>(1227, 2332)	0.228<br>(0.065, 0.390)
Poland	High-middle SDI	3.232<br>(2.693, 3.806)	2283<br>(1895, 2685)	1.593<br>(1.332, 1.855)	3.406<br>(1.625, 5.186)	3080<br>(2459, 3701)	0.974<br>(0.941, 1.007)
Portugal	High-middle SDI	1.720<br>(1.542, 1.905)	428<br>(381, 476)	0.531<br>(0.266, 0.797)	1.784<br>(1.082, 2.486)	532<br>(443, 622)	0.326<br>(0.323, 0.328)
Puerto Rico	High SDI	1.134<br>(0.886, 1.419)	90<br>(70, 112)	-1.735<br>(-2.026, -1.443)	1.223<br>(0.444, 2.003)	114<br>(76, 152)	0.234<br>(0.190, 0.278)
Qatar	High SDI	2.327<br>(1.647, 3.136)	10<br>(7, 14)	0.646<br>(0.076, 1.220)	/	/	/
Republic of Korea	High SDI	1.723<br>(1.470, 1.996)	1461<br>(1251, 1691)	-0.409<br>(-0.528, -0.289)	1.651<br>(1.029, 2.273)	2294<br>(1941, 2648)	-0.065<br>(-0.084, -0.046)
Republic of Moldova	High-middle SDI	1.422<br>(1.227, 1.644)	81<br>(70, 93)	1.423<br>(0.724, 2.127)	1.389<br>(0.536, 2.242)	92<br>(64, 120)	-0.246<br>(-0.256, -0.237)
Romania	High-middle SDI	2.401<br>(1.974, 2.892)	889<br>(729, 1072)	1.356<br>(0.739, 1.976)	2.722<br>(1.792, 3.652)	1125<br>(970, 1280)	1.240<br>(1.235, 1.244)
Russian Federation	High-middle SDI	3.918<br>(3.398, 4.505)	9130<br>(7920, 10483)	1.464<br>(0.380, 2.560)	3.846<br>(-3.365, 11.057)	16852<br>(-2253, 35957)	-0.047<br>(-0.090, -0.003)
Rwanda	Low SDI	2.220<br>(1.549, 2.931)	110<br>(76, 147)	-1.929<br>(-2.178, -1.679)	2.460<br>(-0.261, 5.181)	211<br>(113, 309)	1.702<br>(1.568, 1.836)
Saint Kitts and Nevis	High-middle SDI	3.308<br>(2.845, 3.827)	2<br>(2, 2)	-0.134<br>(-0.568, 0.303)	/	/	/
Saint Lucia	Middle SDI	6.244<br>(5.179, 7.408)	13<br>(11, 15)	-0.330<br>(-0.945, 0.288)	/	/	/
Saint Vincent and the	Middle SDI	3.259	4	0.423	/	/	/

Grenadines		(2.798, 3.760)	(4, 5)	(0.237, 0.610)			
Samoa	Middle SDI	3.003<br>(2.258, 3.856)	4<br>(3, 5)	-1.040<br>(-1.129, -0.951)	/	/	/
San Marino	High SDI	2.672<br>(1.770, 3.693)	2<br>(1, 3)	-0.265<br>(-0.435, -0.094)	/	/	/
Sao Tome and Principe	Low-middle SDI	1.677<br>(1.328, 2.063)	2<br>(1, 2)	0.048<br>(-0.178, 0.276)	/	/	/
Saudi Arabia	High-middle SDI	1.002<br>(0.785, 1.261)	140<br>(105, 184)	0.449<br>(0.300, 0.599)	0.704<br>(0.321, 1.088)	228<br>(175, 281)	-0.366<br>(-0.411, -0.320)
Senegal	Low SDI	1.409<br>(1.044, 1.824)	93<br>(69, 121)	-0.714<br>(-0.848, -0.579)	1.801<br>(0.904, 2.698)	139<br>(105, 174)	0.175<br>(0.130, 0.220)
Serbia	High-middle SDI	4.509<br>(3.606, 5.508)	709<br>(560, 873)	1.280<br>(0.910, 1.650)	3.911<br>(2.304, 5.518)	729<br>(598, 860)	-0.735<br>(-0.764, -0.707)
Seychelles	High-middle SDI	1.921<br>(1.485, 2.502)	2<br>(1, 2)	0.007<br>(-0.298, 0.313)	/	/	/
Sierra Leone	Low SDI	1.368<br>(0.932, 1.941)	43<br>(29, 62)	-0.754<br>(-0.904, -0.604)	1.753<br>(0.676, 2.830)	65<br>(43, 87)	1.077<br>(1.004, 1.150)
Singapore	High SDI	2.025<br>(1.718, 2.271)	146<br>(125, 163)	-1.780<br>(-1.910, -1.649)	1.673<br>(0.750, 2.595)	242<br>(178, 305)	-0.881<br>(-0.890, -0.872)
Slovakia	High SDI	2.107<br>(1.658, 2.617)	190<br>(149, 236)	0.713<br>(0.405, 1.022)	1.791<br>(0.697, 2.885)	214<br>(156, 271)	-0.875<br>(-0.905, -0.845)
Slovenia	High SDI	2.691<br>(2.070, 3.506)	122<br>(94, 158)	0.585<br>(0.090, 1.083)	2.353<br>(0.674, 4.032)	145<br>(95, 196)	-0.272<br>(-0.298, -0.245)
Solomon Islands	Low SDI	3.281<br>(2.064, 5.030)	8<br>(5, 14)	-0.308<br>(-0.427, -0.189)	/	/	/
Somalia	Low SDI	1.857	101	-1.719	1.781	144	0.441

		(0.766, 2.907)	(41, 167)	(-1.790, -1.648)	(0.775, 2.787)	(106, 181)	(0.408, 0.474)
South Africa	Middle SDI	2.091<br>(1.872, 2.292)	816<br>(733, 897)	-1.497<br>(-1.857, -1.136)	2.599<br>(0.328, 4.870)	1040<br>(683, 1397)	-0.049<br>(-0.166, 0.068)
South Sudan	Low SDI	1.799<br>(1.148, 2.619)	57<br>(36, 85)	-1.751<br>(-1.859, -1.642)	1.115<br>(0.162, 2.068)	68<br>(38, 98)	-0.263<br>(-0.387, -0.138)
Spain	High-middle SDI	2.295<br>(2.042, 2.549)	2402<br>(2107, 2684)	0.281<br>(0.093, 0.469)	2.273<br>(1.014, 3.532)	3084<br>(2447, 3721)	0.576<br>(0.536, 0.615)
Sri Lanka	High-middle SDI	1.710<br>(1.287, 2.209)	374<br>(280, 489)	-0.168<br>(-0.843, 0.511)	1.551<br>(0.956, 2.146)	540<br>(445, 636)	-0.523<br>(-0.533, -0.513)
Sudan	Low-middle SDI	1.113<br>(0.727, 1.793)	185<br>(117, 297)	0.301<br>(0.248, 0.354)	1.035<br>(0.585, 1.485)	273<br>(222, 325)	0.002<br>(-0.037, 0.041)
Suriname	Middle SDI	2.299<br>(1.912, 2.755)	13<br>(11, 15)	-0.187<br>(-0.752, 0.381)	/	/	/
Sweden	High SDI	4.754<br>(4.257, 5.223)	1134<br>(998, 1256)	-1.241<br>(-1.455, -1.026)	4.715<br>(2.352, 7.079)	1411<br>(1112, 1709)	0.274<br>(0.248, 0.300)
Switzerland	High SDI	2.993<br>(2.594, 3.390)	592<br>(509, 679)	-1.371<br>(-1.605, -1.135)	2.676<br>(1.578, 3.774)	707<br>(578, 837)	-0.721<br>(-0.735, -0.707)
Syrian Arab Republic	Middle SDI	1.179<br>(0.923, 1.526)	113<br>(87, 148)	0.216<br>(0.030, 0.401)	1.379<br>(0.628, 2.130)	248<br>(188, 308)	1.577<br>(1.570, 1.584)
Taiwan (Province of China)	High SDI	2.794<br>(2.220, 3.538)	1099<br>(872, 1389)	2.598<br>(2.276, 2.920)	2.924<br>(1.331, 4.517)	1673<br>(1346, 2000)	0.876<br>(0.814, 0.938)
Tajikistan	Low-middle SDI	3.371<br>(2.809, 4.056)	106<br>(86, 129)	1.415<br>(0.915, 1.918)	3.681<br>(1.103, 6.258)	188<br>(130, 247)	0.891<br>(0.828, 0.954)
Thailand	Middle SDI	1.957<br>(1.469, 2.483)	1893<br>(1419, 2403)	-0.485<br>(-0.882, -0.087)	2.251<br>(1.295, 3.206)	3305<br>(2777, 3833)	1.135<br>(1.109, 1.160)
Timor-Leste	Low-middle SDI	1.455	10	1.781	/	/	/

		(1.033, 1.981)	(7, 13)	(1.670, 1.892)			
Togo	Low SDI	1.646<br>(1.315, 2.116)	51<br>(39, 67)	-0.765<br>(-0.924, -0.606)	2.090<br>(0.861, 3.319)	83<br>(58, 109)	1.040<br>(1.007, 1.073)
Tokelau	Middle SDI	2.986<br>(2.411, 3.742)	0<br>(0, 0)	-0.610<br>(-0.655, -0.565)	/	/	/
Tonga	Middle SDI	2.062<br>(1.668, 2.572)	2<br>(1, 2)	0.260<br>(-0.048, 0.569)	/	/	/
Trinidad and Tobago	High-middle SDI	4.363<br>(3.258, 5.640)	78<br>(58, 102)	-0.067<br>(-0.395, 0.264)	4.574<br>(1.683, 7.465)	110<br>(75, 146)	0.577<br>(0.517, 0.637)
Tunisia	Middle SDI	1.121<br>(0.829, 1.492)	129<br>(95, 173)	0.204<br>(0.100, 0.308)	1.175<br>(0.630, 1.720)	190<br>(147, 233)	0.350<br>(0.333, 0.367)
Turkey	High-middle SDI	2.081<br>(1.664, 2.573)	1803<br>(1439, 2236)	-1.166<br>(-1.467, -0.864)	1.927<br>(1.048, 2.805)	2244<br>(1901, 2586)	-1.278<br>(-1.286, -1.269)
Turkmenistan	Middle SDI	2.345<br>(1.904, 2.904)	83<br>(67, 104)	2.827<br>(2.360, 3.295)	2.997<br>(1.352, 4.643)	167<br>(125, 210)	2.910<br>(2.876, 2.944)
Tuvalu	Low-middle SDI	3.520<br>(2.704, 4.601)	0<br>(0, 0)	-0.758<br>(-0.847, -0.668)	/	/	/
Uganda	Low SDI	2.067<br>(1.263, 2.765)	250<br>(155, 342)	-0.744<br>(-0.849, -0.638)	2.629<br>(1.458, 3.800)	363<br>(298, 429)	-0.502<br>(-0.513, -0.491)
Ukraine	High-middle SDI	2.750<br>(2.307, 3.269)	2028<br>(1701, 2407)	1.629<br>(0.077, 3.205)	3.353<br>(0.093, 6.614)	2810<br>(1800, 3819)	3.042<br>(2.801, 3.283)
United Arab Emirates	High SDI	2.034<br>(1.076, 3.321)	76<br>(37, 133)	0.058<br>(-0.684, 0.806)	1.783<br>(0.489, 3.077)	180<br>(116, 244)	2.768<br>(2.668, 2.868)
United Kingdom	High SDI	5.896<br>(5.331, 6.222)	8431<br>(7552, 8921)	-1.650<br>(-1.716, -1.585)	6.182<br>(3.200, 9.163)	11369<br>(9186, 13552)	1.571<br>(1.499, 1.644)
United Republic of Tanzania	Low SDI	2.671	566	-0.939	4.052	842	-0.446

		(1.716, 3.765)	(368, 803)	(-1.082, -0.795)	(2.391, 5.714)	(711, 973)	(-0.462, -0.429)
United States of America	High SDI	2.403<br>(2.201, 2.550)	13940<br>(12628, 14828)	-2.502<br>(-2.680, -2.324)	2.877<br>(1.671, 4.083)	20880<br>(17843, 23916)	2.133<br>(2.050, 2.216)
United States Virgin Islands	High-middle SDI	3.560<br>(3.039, 4.161)	7<br>(6, 8)	0.134<br>(-0.245, 0.515)	/	/	/
Uruguay	High-middle SDI	4.755<br>(4.297, 5.208)	267<br>(242, 292)	-0.204<br>(-0.534, 0.126)	4.466<br>(2.192, 6.741)	300<br>(233, 368)	0.267<br>(0.233, 0.301)
Uzbekistan	Middle SDI	1.823<br>(1.561, 2.092)	237<br>(197, 282)	3.905<br>(3.581, 4.230)	1.599<br>(0.857, 2.341)	532<br>(429, 636)	3.020<br>(2.988, 3.052)
Vanuatu	Low-middle SDI	3.375<br>(2.280, 4.678)	5<br>(3, 7)	-0.199<br>(-0.350, -0.048)	/	/	/
Venezuela (Bolivarian Republic of)	Low-middle SDI	2.314<br>(1.756, 2.979)	631<br>(478, 818)	0.281<br>(-0.864, 1.440)	3.713<br>(1.121, 6.305)	1438<br>(1027, 1849)	4.268<br>(4.192, 4.345)
Viet Nam	Middle SDI	1.804<br>(1.384, 2.245)	1440<br>(1088, 1814)	0.590<br>(0.548, 0.632)	1.678<br>(1.172, 2.184)	2202<br>(1944, 2460)	0.487<br>(0.475, 0.499)
Yemen	Low SDI	1.131<br>(0.792, 1.641)	131<br>(89, 195)	0.458<br>(0.374, 0.543)	1.442<br>(0.664, 2.220)	214<br>(165, 262)	0.872<br>(0.852, 0.892)
Zambia	Low-middle SDI	3.238<br>(2.464, 4.228)	181<br>(137, 242)	0.364<br>(0.217, 0.511)	5.403<br>(2.675, 8.130)	385<br>(307, 463)	2.631<br>(2.608, 2.654)
Zimbabwe	Low-middle SDI	2.394<br>(1.895, 3.050)	154<br>(119, 199)	0.416<br>(0.199, 0.634)	2.748<br>(1.358, 4.139)	234<br>(183, 285)	0.723<br>(0.670, 0.776)

ASMR, age-standardized mortality rate; AAPC, average annual percent change; SDI, socio-demographic index; UI, uncertainty interval; CI, confidence interval.
